# Evidence-Based Guidelines and Clinical Pathways in Stroke Rehabilitation—An International Perspective

**DOI:** 10.3389/fneur.2019.00200

**Published:** 2019-03-08

**Authors:** Thomas Platz

**Affiliations:** ^1^Spinal Cord Injury Unit, Centre for Neurorehabilitation, Intensive and Ventilation Care, BDH-Klinik Greifswald, University of Greifswald, Greifswald, Germany; ^2^Special Interest Group Clinical Pathways, World Federation for NeuroRehabilitation, North Shields, United Kingdom

**Keywords:** stroke, rehabilitation, guideline, practice recommendation, evidence

## Abstract

A high societal burden and a considerable increase in stroke-related disability was globally observed over the last 3 decades, and is expected to continue implying a major challenge for societies around the word. Structured multidisciplinary stroke rehabilitation reduces stroke-related disability both in older and younger stroke survivors of either sex and independent of stroke severity. In addition, there is rapidly increasing evidence to support the clinical effectiveness of specific stroke rehabilitation interventions. Evidence-based guidelines help to promote best possible clinical practice. Inherent difficulty for their provision is that it takes enormous efforts to systematically appraise the evidence for guidelines and their regular updates, if they should not be at risk of bias by incomplete evidence selection. A systematic review of the pertaining literature indicates that the currently published stroke rehabilitation guidelines have a national background and focus and represent the health care situations in high-income countries. Societies around the globe would benefit from central evidence sources that systematically appraise the available evidence and make explicit links to practice recommendations. Such knowledge could facilitate a more wide-spread development of valid comprehensive up-to-date evidence-based national guidelines. In addition, the development of genuine international evidence-based stroke rehabilitation guidelines that focus on therapeutic approaches rather than organizational issues, could be used by many to structure regional or local stroke rehabilitation pathways and to develop their resources in a way that will eventually achieve effective stroke rehabilitation. Such international practice recommendations for stroke rehabilitation are currently under development by the World Federation for NeuroRehabilitation (WFNR).

## Global Burden of Disease and Stroke-Related Disability

Preventive measures and improved health care led to a decrease of age-standardized stroke mortality rates over the last few decades, while the absolute number of people affected per year by a new stroke, stroke-related deaths, and the number of stroke survivors living in our societies considerably increased leading to a growing burden of disease and related disability (1). From 1990 to 2010 mortality rates decreased in high-income countries (−37%, 95% confidence interval [95% CI] −31 to −41%) and in low- and middle-income countries (−20%, 95% CI −15 to −30%). In the same time stroke-related deaths (absolute number), number of new stroke survivors, number of stroke survivors living in the society, and lost disability-adjusted life-years all increased (on average by +26, +68, +84, +12%, respectively). Similarly, the Global Burden of Disease Study 2015 group reported an increase of ischemic stroke prevalence (number of stroke survivors living in societies) by 21.8% from 2005 to 2015 (i.e., from 20 467.3 to 24 929.0 thousands) and of years lived with disability by 22.0% (i.e., from 2 999.9 to 3 659.9 thousands) during that time (2).

With the demographic developments to be foreseen (population on average growing older in many countries or less dying from communicable diseases) these trends will continue and societies around the globe are well-advised to plan their health-care resources and societal efforts to cope with the increase in neuro-disabilities efficiently.

## Effectiveness of Stroke Rehabilitation

Both stroke prevention and effective stroke rehabilitation can decrease the burden of stroke-relating disabilities. This review focuses on options offered by stroke rehabilitation and ways to promote its effectiveness through evidence-based guidelines. At a regional or local level such guidelines can be implemented by clinical pathways, i.e., structured, multidisplinary, and multi-step plans of care that then facilitate effective stroke rehabilitation.

Indeed, dedicated care in multidisciplinary stroke units leads to higher rates of independence with activities of daily living (ADL) and results in less need to receive long-term institutional care after stroke (3). In this Cochrane review, a meta-analysis including 21 randomized controlled trials (RCTs) with a total of 39,994 participants showed a reduced rate of death or institutionalized care (OR 0.78, 95% CI 0.68 to 0.89; *P* = 0.0003) and death or dependence (OR 0.79, 95% CI 0.68 to 0.90; *P* = 0.0007) after stroke unit care compared to care in general wards post stroke, without significantly increasing length of stay, and independent of age, sex, or stroke severity.

In addition, it could be shown that specific interventions for stroke rehabilitation promote functional recovery and reduce disability: Both arm-robot therapy and mirror therapy have robustly shown to reduce motor deficits and enhance arm function (4, 5). Similarly, the use of electro-mechanical gait training increases the number of stroke patients that re-gain the ability to walk (6) and the use of treadmill training (with partial body-weight-support) helps to improve walking speed and walking endurance among ambulatory stroke survivors (7).

Thus, contingent to the availability of multidisciplinary specialized stroke services, knowledge about effective rehabilitation therapies (evidence), and both the skill and resources to apply them in clinical practice stroke-related disability can effectively be reduced among stroke survivors world-wide.

## Evidence-Based Stroke Rehabilitation, Obstacles for Implementation, and Guidance by Practice Recommendations

Necessary health care structures for stroke rehabilitation are, however, not available in many countries. Stroke service teams integrate aside from specialist doctors and nurses various therapeutic professions such as physiotherapy, occupational therapy, speech and language therapy, (neuro)psychology, and social workers to name just a “core set” of professions.

The density of physiotherapist available in high-income countries is more than 900 per 1 million inhabitants while below 25 in Africa; the corresponding figures for occupational therapists are more than 400 per 1 million inhabitants in high-income countries vs. < 15 per 1 million inhabitants in Africa; and there are basically no speech and language therapists available in most African countries while high-income countries such as USA or Australia have more than 300 per 1 million (8). Lack of resources is prevailing in many other countries to a varying extent (8).

Another issue for best clinical practice is that of knowledge management. The number of published clinical research (clinical trials) directly applicable to clinical practice is rapidly expanding making it more and more difficult, if not impossible for the individual health care professional to keep up-to-date with the existing evidence.

**Figure 1:**
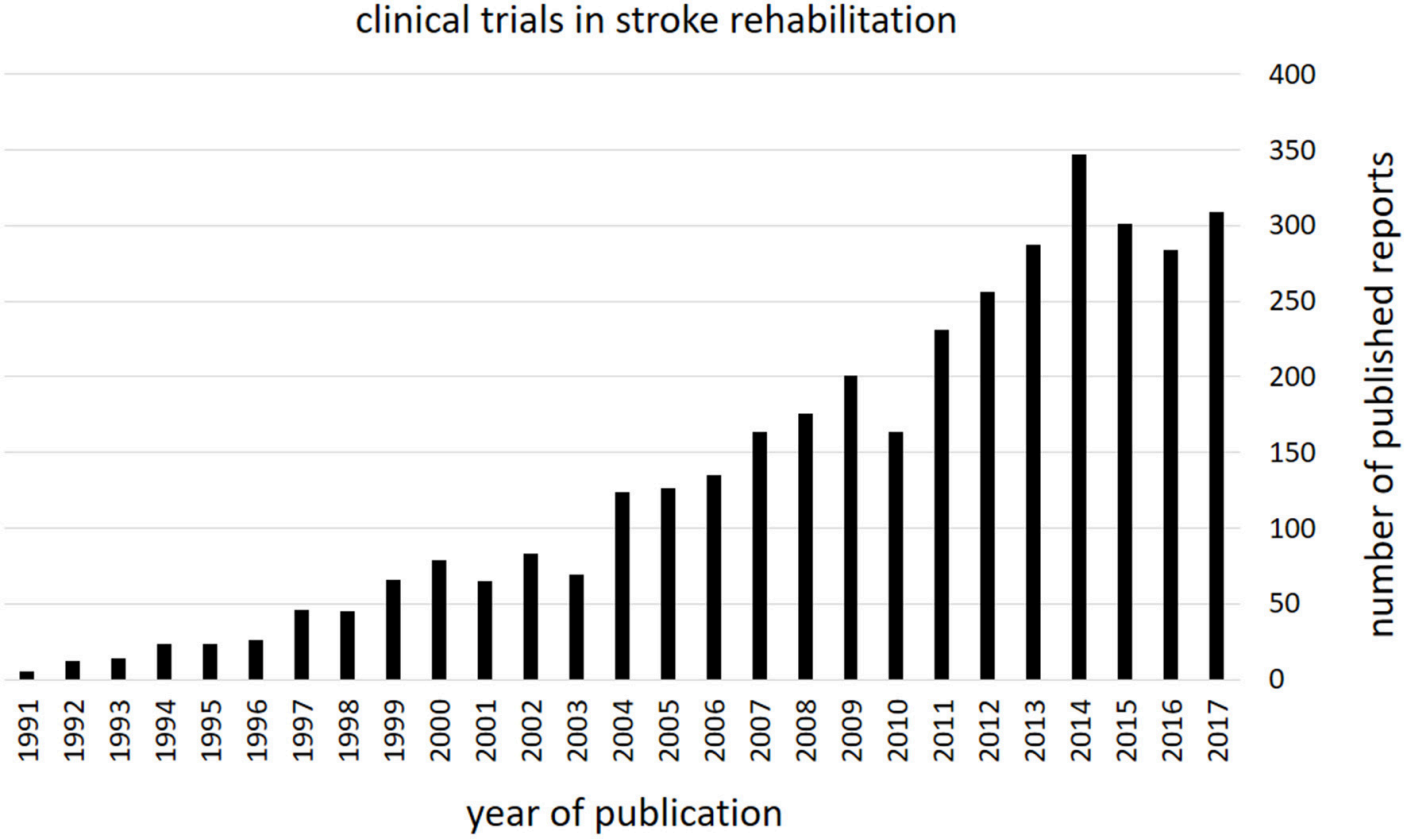
Stroke rehabilitation—clinical trial publications. The figure shows the number of clinical trials reports per year as listed by PubMed (retrieved from PubMed from https://www.ncbi.nlm.nih.gov/pubmed on 20.11.2018). Note the considerable increase in evidence that became available over the last three decades.

Figure 1 illustrates a steep rise in the number of clinical trial reports on “stroke rehabilitation” listed by PubMed from 1991 to 2017. How should a health care professional be able to search, obtain, critically appraise and synthesize all the evidence that's becoming available each year?

Systematic reviews like Cochrane reviews help to provide a balanced, valid, and mostly up-to-date picture of the available external evidence. They are, however, restricted to only a limited number of health care questions addressed. Thus, while they give a valuable orientation for some topics they are not available for many others. Furthermore, they provide a picture of the evidence, but do refrain from making explicit clinical practice recommendations leaving the reader with a degree of uncertainty how to apply the knowledge.

Evidence-based clinical practice guidelines are meant to provide this guidance. If they are comprehensive, covering a broad range of topics in stroke rehabilitation and are evidence-based they are both valid and clinically useful.

## Existing Guidelines for Stroke Rehabilitation

### Objective

For this review, a systematic search for and an appraisal of stroke rehabilitation guidelines was performed. The objectives were to document the existing guidelines, to distinguish between guidelines that were general stroke guidelines with a rehabilitation section, a genuine, yet broad stroke rehabilitation guideline, a guideline that addresses a specific topic within stroke rehabilitation (e.g., mobility), or a guideline that focuses on a specific profession involved in stroke rehabilitation (such as physiotherapy), to classify them as consensus-based (consensus process within guideline development group) and/or evidence-based (systematic search and critical appraisal of the literature), as national or international (based on their primary intention and target user group), and to document the date when the last update has been published. Guidelines were eligible if published within the last 10 years (year of publication 2009 to 2018), older guidelines were no longer considered relevant for clinical practice.

Based on the retrieved guidelines a qualitative synthesis in terms of their suitability for an international context is attempted.

### Systematic Search and Selection of Guidelines

A search for stroke rehabilitation guidelines was performed on 16.11.2018 in three electronic databases, i.e., PubMed (https://www.ncbi.nlm.nih.gov/pubmed), AskDoris (www.askdoris.org), and Guideline International Network (www.g-i-n.net). The database DORIS was developed by the Cochrane Stroke Group and provides easy access to evidence-based stroke research and to a limited extent to stroke-related guidelines. The Guidelines International Network hosts an extensive library for guidelines that is accessible online. The search algorithm used for PubMed was (“stroke rehabilitation”[MeSH Terms] OR (“stroke”[All Fields] AND “rehabilitation”[All Fields]) OR “stroke rehabilitation”[All Fields]) AND (Practice Guideline[ptyp] AND “2008/11/19”[PDat]: “2018/11/16”[PDat]) and was adapted for the other databases. A hand search based on the material retrieved (e.g., references) amended the search process.

After removal of duplicates the electronic entries were screened for relevance by title and abstract, the remaining entries were critically appraisal for selection and contents based on full-text review.

### Results

Forty-nine entries of publications (three of them were obtained by hand search) remained after removal of duplicates.

Of those, 31 entries were excluded for the following reasons [number of entries]: no guideline [2]; guideline published before 2009 [1]; meanwhile updated [1]; not disease-related (general exercise standards, critical care patients) [2]; addressing other or various diseases (CMD, CVD, chronic heart failure, dyslipidaemia), not specifically stroke [5]; for disease prevention [7]; acute care (primary stroke center, telestroke, atrial fibrillation, intracerebral hemorrhage management [2], subarachnoidal hemorrhage management) [7]; covering other specific aspects of stroke management (organization of services, transition between health care segments etc.) [4]; 1 registry recommendation [1]; guideline for research [1].

Eighteen guideline or practice recommendation publications were selected (9–26); their characteristics are presented in Table 1.

**Table 1:** Characteristics of guidelines and practice recommendations related to stroke rehabilitation (published from 2009 to 2018).

**Author**	**Geography**	**Date**	**Language**	**General (including rehabilitation)**	**Rehabilitation**	**Topic**	**Profession**	**Consensus**	**Evidence**
RCP (9)	U.K.	2016	English	+				+	+
SASS (10, 11)	South Africa	2010, 2011	English	+				+	
SF (AUS) (12)	Australia	2017	English	+				+	+
AHA/ASA (13)	U.S.A.	2016	English		+			+	+
CSBPR (14)	Canada	2016	English		+			+	+
NCGC (15)	U.K.	2013	English		+			+	+
SIGN (16)	Scotland	2010	English		+			+	+
VA/DoD (17)	U.S.A.	2010	English		+			+	+
ABMFR (18)	Brasilia	2012	Portuguese			+ (Motor)			+
HAS (19)	France	2012	French			+ (Motor)		+	+
DGNR (20)	Germany	2009	German			+ (Arm paresis)		+	+
DGNR (21)	Germany	2015	German			+ (Mobility)		+	+
AHA/ASA (22)	U.S.A.	2014	English			+ (Physical activity)		+	+
AHA/ASA (23, 24)	U.S.A.	2009	English			+ (Telemedicine)		+	+
KNGF (25)	NL	2014	English, Netherlands				+ (PT)	+	+
AOTA (26)	U.S.A.	2015	English				+ (OT)	+	+

*Author, society or authority issuing the guideline or practice recommendations (reference number in reference list); geography, national or international guideline based on their country of origin and target region; date, date when the last update has been published; general, general stroke guidelines with a rehabilitation section; rehabilitation, genuine, yet thematically broad stroke rehabilitation guideline; topic, a guideline that addresses a specific topic within stroke rehabilitation (e.g., mobility); profession, a guideline that focusses on a specific profession involved in stroke rehabilitation (such as physiotherapy); consensus, consensus-based (i.e., consensus process established within guideline development group); evidence, evidence-based with systematic search and critical appraisal of the literature; ABMFR, Associação Brasileira de Medicina Física e Reabilitação (BR); AHA/ASA, American Heart Association/American Stroke Association (U.S.A.); AOTA, American Occupational Therapy Association (U.S.A.); CSBPR, Canadian Stroke Best Practice Recommendations (CAN); DGNR, Deutsche Gesellschaft für Neurorehabilitation (D); HAS, Haute Autorité de Santé (F); KNGF, Koninklijk Nederlands Genootschap voor Fysiotherapie (NL); NCGC, National Clinical Guideline Centre (U.K.); RCP, Royal College of Physicians (U.K.); SASS, South African Stroke Society (ZA); PT, Physiotherapy; OT, Occupational therapy*.

### Summary and Discussion of Findings

General stroke care guidelines have the advantage that rehabilitation recommendations are linked to the overall stroke management from the acute care to long-term support (9–12). The Royal College of Physicians (RCP) guideline (9) makes explicit statements regarding both organizational aspects, specific treatment aspects (focus on ADL, arm function, mobility cognition, communication, and other aspects), and in terms of commissioning stroke rehabilitation.

The Australian guideline for stroke management (12) is similarly broad in scope. When it comes to rehabilitation, a specific chapter makes recommendations for interventions targeting impairments (sensorimotor, communication and cognitive) and activities. Another chapter on “managing complications” addresses secondary impairments or complications (i.e., impairments that result from the primary impairments). Aspects of care related to participation and reintegration into the community, including self-management are provided in a chapter on “community participation and long-term care.”

The comprehensiveness of these guidelines (9–12) is a major strength for anyone who wants to build clinical pathways for stroke rehabilitation in a specific regional or local health care situation.

Only one of the 8 general stroke or stroke rehabilitation guidelines (9–17) comes, however, from a low- or middle-income country, i.e., from South Africa (10, 12). Its major advantage is, that it explicitly takes the regional “underresourced setting” into account. All the other guidelines from the high-income countries cannot easily be applied in a situation like in South Africa where there is little specialized stroke health care in rural parts of the country.

National guidelines that primarily focus on stroke rehabilitation (13–17) can equally provide comprehensive guidance on both organization and content issues relevant for stroke rehabilitation, and they also provide answers that are adjusted to the regional health care system. As an example, the U.S. stroke rehabilitation guideline (13) explicitly takes the situation into account where immediately after a short acute care treatment intensive rehabilitation care is provided in inpatient rehabilitation facilities (IRFs), followed by skilled nursing facilities (SNFs), that provide “subacute” rehabilitation, yet without daily supervision by a physician, and other care structures available in the U.S. Therefore, the content of these guidelines has restricted validity outside their context, especially when health care system and organizational aspects are addressed.

Some stroke rehabilitation guidelines are structured to answer clinical questions. An example from National Clinical Guideline Centre (15) is “In people after stroke what is the clinical and cost-effectiveness of repetitive task training vs. usual care on improving function and reducing disability?” The reported evidence provided for arm rehabilitation (4 RCTs) is not conclusive. The recommendation given is “Offer people repetitive task training after stroke on a range of tasks for upper limb weakness (such as reaching, grasping, pointing, moving, and manipulating objects in functional tasks).” Before this guideline was published a Cochrane Review (27) came, however, to the conclusion that “Repetitive task training resulted in modest improvement across a range of lower limb outcome measures, but not upper limb outcome measures.” While the “clinical question approach” can certainly be useful, it carries a risk for lack of scope, e.g., not simultaneously looking at the diverse other forms of arm rehabilitation therapies, and to skip relevant (and more effective) treatment options. Indeed, another stroke rehabilitation guideline from the U.K. (16) that more comprehensively looked into arm rehabilitation techniques came to a different conclusion and recommended with the highest level (A) “Repetitive task training is not routinely recommended for improving upper limb function.”

An observation made with the general stroke and stroke rehabilitation guidelines is that the evidence integrated in the guideline development process varies considerably and (even when systematic) is frequently limited. As an example these guidelines list from 1 (10), 8 (9), 10 (12), 33 (16) to 82 (13) references for their arm rehabilitation recommendations while more than 400 RCTs and more than 100 systematic reviews (SRs) were published for arm rehabilitation post stroke until mid of 2017 [own systematic search for guideline development; work in progress, update of (20)]. There is thus a risk of bias by evidence selection. And that risk might increase with the overall spectrum that a guideline intends to cover.

With thematically more focused guidelines addressing a function (18–22) or a profession (25, 26) in stroke rehabilitation, it is easier to provide a comprehensive critical appraisal of the pertaining clinical research evidence (e.g., as in 20–26). Thereby, the chance to promote recommendations that reflect the best available external evidence at the time of their development is increased. Their development can, however, consume a lot of resources when a substantial evidence-base is available while they contribute only to a single topic in stroke rehabilitation. An inherent problem is that it is difficult to provide the resources for their development and hence to keep them updated. Further, it would not be economical to reproduce the work for such an intensive evidence-based guideline development in each country. And therefore, the reliance of guideline developers on the most relevant SRs is a valid pragmatic approach, but does—as illustrated above—imply risk of bias by evidence selection.

Limitation of the review: The reported electronic search for stroke rehabilitation guidelines might not have detected all guidelines available [e.g., missed Scandinavian guidelines (28, 29)]. The coverage was, however, representative and complete enough to address the relevant issues for this review.

## A New Format of Evidence Synthesis to Link Best Evidence to Practice Recommendations

Guideline and pathway developers might benefit from an initiative to generate (central) sources that bridge comprehensive up-to-date external evidence with clinical practice recommendations, a task that still needs to be structurally solved in the future.

The SIG Clinical Pathways of the World Federation for NeuroRehabilitation (WFNR) is currently developing and testing such an approach for domains where systematic reviews based on RCTs are available (30). The concept is to comprehensively search for systematic reviews addressing therapeutic effects for a given clinical problem (e.g., any intervention for post stroke cognitive impairment), select the most informative and valid up-to-date systematic reviews for critical appraisal and data extraction with an outcome-centered approach, followed by a structured multi-step approach from the evidence to practice recommendations. This approach avoids potential pitfalls of narrowness of health care questions, reduces workload for critical appraisal by selecting the most relevant systematic reviews and does not end with the presentation of evidence (as systematic reviews do), but explicitly links the evidence to practice recommendations in a systematic transparent way. Such scientific information could then easily be adopted as reference by guideline developers worldwide.

“Central” guidance might also be warranted for domains where the evidence is limited to (mainly) observational studies and the generation of a methodologically and clinically valid link between evidence and clinical practice recommendation is challenging.

## Practice Recommendations and Clinical Pathways—Global and Local Aspects

Stroke care in accordance with evidence-based stroke rehabilitation guidelines is effective and can reduce the burden of stroke-related disabilities. Yet, in most countries national evidence-based stroke rehabilitation guidelines are not available, nor is their wide-spread development a realistic scenario. And, as stated above the usefulness of guidelines developed in high-income countries is limited for low- and middle-income countries.

In that situation, international stroke rehabilitation guidelines would be helpful for a broader readership if they were not only applicable to a specific regional setting, but could provide guidance for professionals from diverse health care system backgrounds. One way to achieve that goal is when guidelines address the health care questions for stroke rehabilitation in a generic way, independent of organizational and resource settings, i.e., based on stroke sequelae in terms of impairments and activity limitations. If they provided the best available external evidence and hence evidence-based recommendations for treatment of main functional stroke sequelae such as dysphagia, arm dysfunction, mobility deficits, perceptual, communication, cognitive, behavioral and emotional disorders they could provide guidance for effective stroke rehabilitation without being bound to organizational pre-requisites. Such guidance does not solve the resource problem, but does nevertheless help to make best use of the resources available. In addition, it can promote the development of regional organizational settings and resources in a way that best supports effective stroke rehabilitation.

Regional or local clinical pathways for stroke rehabilitation could make use of these international practice recommendations and implement them in a way that is achievable in the local situation. With high level consensus- and evidence-based recommendations being provided centrally all that remains to be done at a local level is to build contextualized clinical pathways, their communication, implementation, evaluation, and adjustment. These more confined goals might be easier to achieve and in addition the solutions more meaningful by their suggested contextualization, especially for low- or middle-income countries.

In that way, international stroke rehabilitation guidelines could generate a broad impact without the need for each country to invest time and effort to generate their own evidence-based guidelines. Such international stroke rehabilitation guidelines are currently developed by the WFNR and are intended to be published open-access.

The work will also support the WHO initiative “Rehabilitation 2030” and its working group that identifies evidence-based rehabilitation interventions suitable for implementation in low and middle income countries with stroke being a prioritized area (31).

## Conclusions

People around the globe can make use of the guidance that is available from existing stroke guidelines, both in terms of service set-up and organization as well as on how to therapeutically address specific problem that people are faced with after stroke. Two strategies have been adopted, the generation of general stroke or stroke rehabilitation guidelines (9–17) with a risk of bias by evidence selection, and a more focal approach (18–26) with a higher chance of complete evidence coverage, yet restricted thematic scope and difficulties to keep them up-to-date. Hence, the international community of guideline developers could benefit from a centrally available source of evidence synthesis (that goes beyond SRs) with an explicit link to practice recommendations.

Further, a characteristic of the available guidelines is their national focus and their representation of health care situations in high-income countries. Accordingly, they are of limited applicability in other, especially low- and middle-income countries.

Since the development of guidelines for each country is not a realistic scenario while the adherence to evidence-based stroke rehabilitation guidelines is likely to reduce the burden of stroke-related disability in societies, a pragmatic solution could be to develop international stroke rehabilitation guidelines.

These could then regionally or locally be used to both generate contextualized stroke rehabilitation pathways based on the resources locally available and to develop the organization of health care and related resources in a way that will eventually promote effective stroke rehabilitation. That being achieved any benefit for stroke survivors could further be enhanced by additional implementation strategies such as education and print material for both professionals and stroke survivors (32).

## Author Contributions

The author confirms being the sole contributor of this work and has approved it for publication.

### Conflict of Interest Statement

The author declares that the research was conducted in the absence of any commercial or financial relationships that could be construed as a potential conflict of interest.
